# CM1, a Chrysin Derivative, Protects from Endotoxin-Induced Lethal Shock by Regulating the Excessive Activation of Inflammatory Responses

**DOI:** 10.3390/nu16050641

**Published:** 2024-02-25

**Authors:** Jae-Hyung Lee, Young-Bok Ko, Yong-Min Choi, Jinju Kim, Hwan-Doo Cho, Hyeonil Choi, Ha-Yeon Song, Jeong-Moo Han, Guang-Ho Cha, Young-Ha Lee, Jin-Man Kim, Woo-Sik Kim, Eui-Baek Byun, Jae-Min Yuk

**Affiliations:** 1 Department of Infection Biology, College of Medicine, Chungnam National University, Daejeon 35015, Republic of Korea; dlwogud1003@naver.com (J.-H.L.); ymcdragon@naver.com (Y.-M.C.); w1l2s3@naver.com (J.K.); johwandoo@naver.com (H.-D.C.); perfectviewman@naver.com (H.C.); gcha@cnu.ac.kr (G.-H.C.); yhalee@cnu.ac.kr (Y.-H.L.); 2 Infection Control Convergence Research Center, College of Medicine, Chungnam National University, Daejeon 35015, Republic of Korea; jinmank@cnu.ac.kr; 3 Department of Medical Science, College of Medicine, Chungnam National University, Daejeon 35015, Republic of Korea; koyoung27@gmail.com; 4 Department of Obstetrics & Gynecology, Chungnam National University Hospital, Daejeon 35015, Republic of Korea; 5 Korea Advanced Radiation Technology Institute, Korea Atomic Energy Research Institute, Jeongeup 56212, Jeonbuk, Republic of Korea; songhy@kaeri.re.kr (H.-Y.S.); jmhahn@kaeri.re.kr (J.-M.H.); 6 Department of Pathology, College of Medicine, Chungnam National University, Daejeon 35015, Republic of Korea; 7 Functional Biomaterial Research Center, Korea Research Institute of Bioscience and Biotechnology, Jeongeup 56212, Jeonbuk, Republic of Korea

**Keywords:** sepsis, inflammation, Toll-like receptor 4, CM1, TNFAIP3, sirtuin 1

## Abstract

Sepsis, a leading cause of death worldwide, is a harmful inflammatory condition that is primarily caused by an endotoxin released by Gram-negative bacteria. Effective targeted therapeutic strategies for sepsis are lacking. In this study, using an in vitro and in vivo mouse model, we demonstrated that CM1, a derivative of the natural polyphenol chrysin, exerts an anti-inflammatory effect by inducing the expression of the ubiquitin-editing protein TNFAIP3 and the NAD-dependent deacetylase sirtuin 1 (SIRT1). Interestingly, CM1 attenuated the Toll-like receptor 4 (TLR4)-induced production of inflammatory cytokines by inhibiting the extracellular-signal-regulated kinase (ERK)/MAPK and nuclear factor kappa B (NF-κB) signalling pathways. In addition, CM1 induced the expression of TNFAIP3 and SIRT1 on TLR4-stimulated primary macrophages; however, the anti-inflammatory effect of CM1 was abolished by the siRNA-mediated silencing of *TNFAPI3* or by the genetic or pharmacologic inhibition of SIRT1. Importantly, intravenous administration of CM1 resulted in decreased susceptibility to endotoxin-induced sepsis, thereby attenuating the production of pro-inflammatory cytokines and neutrophil infiltration into the lung compared to control mice. Collectively, these findings demonstrate that CM1 has therapeutic potential for diverse inflammatory diseases, including sepsis.

## 1. Introduction

Sepsis is a life-threatening condition that can lead to organ failure in association with high mortality and morbidity, particularly in intensive care units. Sepsis is characterised by excessive systemic inflammation that occurs through uncontrolled and abnormal innate immune responses during overwhelming microbial infections [1,2,3]. Toll-like receptors (TLRs) are pattern recognition receptors (PRRs) that respond to pathogen-associated molecular patterns (PAMPs), which are conserved components of microorganisms [4]. Among these, TLR4 is responsible for sensing lipopolysaccharide (LPS), a component of the outer membrane of Gram-negative bacteria and an important cause of sepsis. TLR4 stimulation initiates the transcriptional upregulation of inflammatory mediators and TIR domain-containing adaptor molecules, such as myeloid differentiation primary-response protein 88 (MyD88) and TIR domain-containing adaptor inducing IFN-β (TRIF) [5].

To prevent the development of autoimmune and inflammatory diseases, TLR signalling pathways are tightly controlled by intracellular mediators. In the TLR4 signalling cascade, the E3 ubiquitin protein ligase TRAF6 regulates the nuclear factor (NF)-κB and mitogen-activated protein kinase (MAPK) signalling pathways, together with the E2 ubiquitin ligase complex of Ubc13 and Uev1A, by catalysing the Lys63-linked polyubiquitination of TRAF6 [6]. Tumour necrosis factor alpha-induced protein 3 (TNFAIP3, A20) is a TNF-induced zinc-finger protein and ubiquitin-editing enzyme with E3 ubiquitin ligase and deubiquitinase activity, and directly inhibits TRAF6 poly-ubiquitination as a negative regulator of TLR signalling [7]. Sirtuin1 (SIRT1) is a nicotinamide adenosine dinucleotide-dependent protein deacetylase that modulates the post-translational activity of histone and non-histone proteins and is implicated in inflammation, apoptosis, metabolism, and the cell cycle [8]. SIRT1 interacts with the NF-κB complex and catalyses deacetylation of the p65/RelA subunit at Lys310, leading to the suppression of NF-κB-dependent transcriptional gene activation [9]. The orphan nuclear receptor oestrogen-related receptor α (NR3B1) negatively regulates TLR-induced inflammation by controlling the transcriptional activation of *Tnfaip3* and SIRT1-mediated suppression of p65 acetylation, leading to enhanced susceptibility to LPS-induced septic shock [10].

Polyphenols are a class of natural compounds found in fruits, vegetables, herbs, and wine; more than 8000 types have been identified to date [11]. Flavonoids are the most abundant type of polyphenols and have been investigated as complementary therapeutics for a variety of diseases [12]. Chrysin, also known as 5,7-dihydroxyflavone, is found in a variety of plants, such as propolis, bitter melon, and walnut pellicle; it exerts antioxidant, anti-inflammatory, and anti-apoptotic effects, but has demonstrated toxicity and safety issues [13,14]. CM1, a hydroxyethyl derivative of chrysin, is generated by the exposure of chrysin methanolic solution to ionising radiation and has an anti-inflammatory effect on primary dendritic cells and dextran sodium salt (DSS)-induced colitis in mice [15]. Moreover, CM1 strongly attenuated TLR4-induced inflammation by upregulating Toll-interacting protein (Tollip) in RAW264.7 macrophages [16]. In this study, we investigated the anti-inflammatory activity of CM1 using chrysin and CM2 as comparators in TLR4-mediated inflammation using primary murine macrophages. We found that TNFAIP3 and SIRT1 are important for the anti-inflammatory effect of CM1 during TLR4 stimulation. In vivo, CM1 showed therapeutic potential for endotoxin-induced septic shock in a mouse model. In summary, CM1 negatively regulated excessive inflammation in vitro in primary macrophages and in vivo in a mouse model of sepsis.

## 2. Materials and Methods

### 2.1. Preparation of CM1 and 2

CM1 and 2, compounds derived from chrysin, were produced using gamma irradiation as described previously [17,18]. To commence the procedure, chrysin (Sigma-Aldrich, St. Louis, MO, USA) was dissolved in methanol to achieve a concentration of 1 mg/mL. This solution was then subjected to gamma irradiation using a ^60^Co source (AECL, IR-79, MDS Nordion International Co., Ltd., Ottawa, ON, Canada) at a dose rate of 10kGy/h, accumulating to a total dose of 100 kGy. Dosimetry for this process was accurately calibrated with the use of 5 mm-diameter alanine dosimeters (Bruker Instruments, Rheinstette, Germany). Following irradiation, the chrysin solution was processed to remove the methanol, which was achieved using a rotary evaporator (RV10, IKA, Maribor, Germany).

### 2.2. Mice

C57BL/6 mice used in our experiments were purchased from Damool Science (Daejeon, Republic of Korea). Additionally, m*Sirt1*^−/−^ mice were generously provided by Prof. Byung-Hyun Park (Biochemistry Department at Chonbuk National University Medical School, Republic of Korea).

### 2.3. Preparation of Cell

Bone marrow-derived macrophages (BMDMs) were isolated from female C57BL/6 mice, aged 5 to 6 weeks. BMDMs underwent differentiation [19] for 5 to 7 days in culture medium enriched with macrophage colony-stimulating factor (M-CSF). In a parallel study, HeLa cells (America Type Culture Collection, Manassas, VA, USA), a human cervical epithelial cancer cell line, were cultured following standard protocols. Both BMDMs and HeLa cells were propagated in Dulbecco’s modified Eagle’s medium (DMEM; Welgene, Gyeongsan, Republic of Korea) fortified with 10% heat-inactivated foetal bovine serum (FBS; Gibco BRL, Waltham, MA, USA) and 1% antibiotic-antimycotic (GibcoTM, Waltham, MA, USA).

### 2.4. Reagents and Antibodies

Chrysin (C80105), Sirtinol (S7942) and EX-527 (E7034) were acquired from Sigma-Aldrich (St. Louis, MO, USA). Lipopolysaccharide (LPS) from Escherichia coli 0111:B4 was procured from InvivoGen (San Diego, CA, USA). For immunoblotting, we used specific antibodies targeting phospho-ERK (4370), phospho-SAPK/JNK (4668), phospho-p38 (9211), IκB-α (9242), phospho-IKKα/β, A20/TNFAIP3 (5630), NF-κB p65 acetyl-K310 (3045), SIRT1 (8469), all sourced from Cell Signalling (Danvers, MA, USA).

### 2.5. Cell Viability Assays

To evaluate the cytotoxicity of chrysin and its derivatives CM1 and CM2 on BMDMs, we employed the Cell Counting Kit-8 (CCK-8) assay (Dojindo Molecular Technologies, Inc., Rockville, MD, USA). We first cultured BMDMs in 96-well plates, following the kit’s guidelines. The cells were exposed to varying doses of chrysin derivatives and incubated for 18 h and then medium was changed with serum-free DMEM containing the CCK-8 reagent. We then measured cell viability by determining the absorbance at 450 nm using a SPECTROstar Nano microplate reader

### 2.6. RNA Extraction, RT-PCR and Real-Time Quantitative PCR

RNA extraction from the activated cells was performed utilizing Trizol reagent (Invitrogen, Waltham, MA, USA), adhering strictly to the guidelines specified by the manufacturer. This extracted RNA was subsequently reverse transcribed into cDNA using a Reverse Transcriptase Premix kit. For further analysis, the resultant cDNA underwent amplification using SolgTM 2X Taq PCR Pre-Mix (Solgent, Daejeon, Republic of Korea). The analysis of the amplified products was conducted in two ways: resolution via 1.5% agarose gel for RT-PCR and real-time quantitative PCR analysis using StepOneTM (Catalog No.4376357) and StepOnePlusTM Software (Version 2.3) (Applied Biosystems, Waltham, MA, USA). Primer sequences utilized in these procedures were as follows: mouse TNFα (forward: 5′-AGCACAGAAAGCATGATCCG-3′; reverse: 5′-CTGATGAGAGGGAGGC CATT-3′), mouse IL-6 (forward: 5′-TACCACTTCACAAGTCGGAGGC-3′; reverse: 5′-CTGCAAGTG CATCATCGTTGTTC-3′), mouse β-actin (forward: 5′-TCATGAAGTGTGACGTTGACATCCGT-3′; reverse: 5′-CCTAGAAGCATTTGCGGTGCACGATG-3′), human TNFAIP3 (forward: 5′-AGAGCAAC TGAGATCGAGCCA-3′; reverse: 5′-CTGGTTGGGATGCTGACACTC-3′), human TNFα (forward: 5′-TCCTCAGCCTCTTCTCCTTCCT-3′; reverse: 5′-ACTCCAAAGTGCAGCAGACAGA-3′), human IL-6 (forward: 5′-GTAGCCGCCCCACACAGA-3′; reverse: 5′-CATGTCTCCTTTCTCAGGGCTG-3′), human β-actin (forward: 5′-CGGAGTCAACGGATTTGGTCGTA-3′; reverse: 5′-AGCCTTCTCC ATGGTGGTGAAGAC-3′).

### 2.7. ELISA

Concentrations of TNFα and IL-6 in mouse serum or cell culture supernatants were determined using ELISA kits (R&D Systems, Minneapolis, MN, USA). Briefly, sandwich ELISA assays were performed according to the protocols provided by the manufacturer. Cytokine concentrations in each sample were measured using standard curves obtained by the dilution of standard protein.

### 2.8. Western Blot Analysis

Protein isolation from cellular lysates was carried out using RIPA buffer supplemented with a freshly prepared protease inhibitor cocktail (Roche Diagnostics GmbH, Mannheim, Germany). Following the extraction, proteins were combined with SDS loading buffer and heated to promote denaturation. Subsequent separation of these proteins was achieved through SDS-PAGE, after which they were transferred to PVDF membranes (Millipore Corp., Billerica, MA, USA). The membranes were then blocked in a 5% non-fat milk solution in TBST for 30 min at room temperature. Primary antibodies, diluted to 1:1000 in 5% BSA-TBST, were applied and incubated for 2 h. After washing off the primary antibodies with TBST, the membranes were incubated with secondary antibodies, diluted at 1:10,000 in milk-TBST, for an additional 2 h. Protein bands were detected using the Immobilon Western HRP Substrate (Merck Millipore, Burlington, MA, USA) with a Fusion Solo System (Vilber Lourmat, Marne-la-Vallee, France), and their intensities were quantified with Image J software (Version 1.8.0).

### 2.9. NF-κB p65 Nuclear Translocation

BMDMs were cultured on 22-mm-diameter glass coverslips in 24-well plates. The cells were exposed to LPS at 100 ng/mL, LPS with chrysin (2.5 µg/mL), or LPS with CM1 (0.1, 0.5, or 2.5 µg/mL) for 30 min. The cells were washed with phosphate-buffered saline (PBS) and fixed in 4% paraformaldehyde for 1 h at room temperature, followed by three washes in PBS with 0.1% Triton X-100 (PBS-T) for 5 min each. Blocking was performed using 10% normal goat serum (Abcam, Cambridge, MA, USA) in PBS for 30 min at ambient temperature. Next, the cells were incubated with primary NF-κB p65 antibody (sc-8008) for 2 h, and then washed and incubated with anti-mouse AlexaFluor 488 secondary antibody for 2 h at room temperature. Subsequently, 4′,6-diamidino-2-phenylindol (DAPI; Sigma-Aldrich, St. Louis, MO, USA) staining was performed for 10 min and stained cells were visualised and imaged using a LSM900 confocal microscope (Zeiss, Oberkochen, Germany).

### 2.10. siRNA Transfection

HeLa cells were subjected to transfection using 30 nM A20-specific siRNA (sc-37655) through the application of Lipofectamine RNAiMAX (Life Technologies Corporation, Carlsbad, CA, USA), following the guidelines provided by the manufacturer. The effectiveness and selectivity of the gene silencing were assessed using RT-PCR and Western blot analyses. Comparative studies were conducted on cells transfected with either the negative control siRNA or siRNA-A20. Post-transfection, these cells exposed to (100 ng/mL) of LPS with CM1 (1 µg/mL) for 18 h.

### 2.11. Mouse Models of Sepsis

Female mice aged between 6 and 8 weeks were selected for experimentation. Before initiating LPS-induced challenge, these mice were administered either sterile PBS or CM1 (at a dosage of 10 mg/kg; 0.2 mg/mouse) intravenously (i.v.) daily over a span of three days. Following this preparatory phase, they were subjected to an intraperitoneal (i.p.) injection of LPS from Escherichia coli O26:B6 (Sigma-Aldrich, St. Louis, MO, USA), at a concentration of 30 mg/kg (0.6 mg/mouse).

### 2.12. Histology and Immunohistochemistry

After euthanasia, spleens, livers, and lungs were swiftly removed from the mice, rinsed in PBS, and subsequently fixed in 10% formalin. Following fixation, these tissues were embedded in paraffin. For histological examination, thin sections (4 μm) were prepared from the paraffin blocks and stained using the haematoxylin and eosin (H&E) method. To specifically assess neutrophil infiltration, the sections underwent a detailed preparation process. Initially, they were deparaffinized and rehydrated via a series of immersions in xylene and graded ethanol solutions (100%, 95%, and 80%), followed by a final rinse in distilled water and PBS for 20 min. Post rehydration, these sections were immunohistochemically stained for neutrophils using the NIMP-R14 antibody (Abcam, Cambridge, MA, USA), to enable detailed analysis.

### 2.13. Statistical Analysis

All experiments were replicated a minimum of three times. For statistical analysis, we used a two-tailed paired Student’s *t*-test to compare mean values and analysed survival data using a log-rank test. Results are reported as means ± standard deviation (SD). Statistical significance was determined using GraphPad Prism v5 software with a threshold of *p* < 0.05.

## 3. Results

### 3.1. Cytotoxic Effects of Chrysin and Its Derivatives CM1 and CM2 in Primary Murine Macrophages

**Figure 1:**
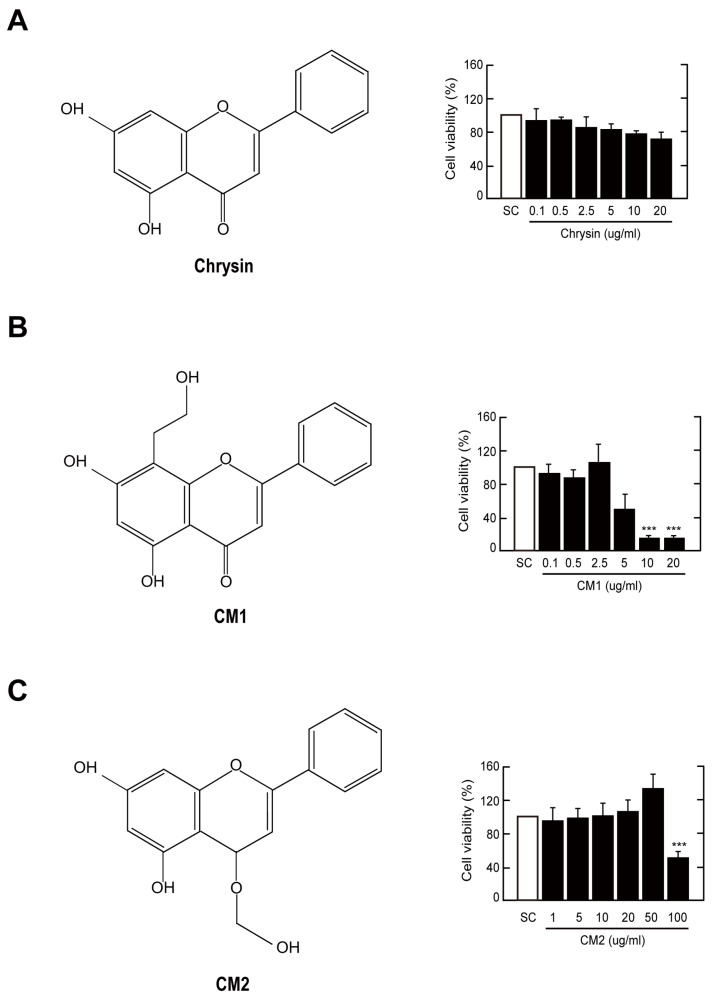
Chemical structures and cytotoxicity in chrysin and its derivatives. (**A**–**C**) BMDMs were incubated with different concentration of chrysin, CM1, or CM2 for 18 h. Cell viability measured by a Cell Counting Kit 8 (CCK-8) assay. Data are representative of three independent experiments and are presented as means ± standard deviation (SD). *** *p* < 0.001, compared with control cells (two-tailed Student’s *t*-test). SC, solvent control (0.01% dimethylsulfoxide).

To investigate the optimal concentrations of chrysin, CM1, and CM2 in terms of toxicity and safety, we evaluated their effects on the viability of BMDMs using a Cell Counting Kit 8 (CCK-8) assay. Chrysin at concentrations of 0.1 to 20 µg/mL did not affect the viability of BMDMs at 18 h (Figure 1A). However, viability was reduced by ~50% (5 µg/mL) and 85% (10 and 20 µg/mL) at 18 h after treatment with CM1 (Figure 1B). Additionally, CM2 at concentrations up to 50 µg/mL did not show cytotoxicity (Figure 1C). Therefore, we used concentrations of 0.1–20 µg/mL chrysin, 0.1–2.5 µg/mL CM1, and 0.1–50 µg/mL CM2 in subsequent experiments.

### 3.2. CM1, but Not Chrysin or CM2, Negatively Regulates TLR4-Induced Production of Inflammatory Cytokines in Primary Murine Macrophages

**Figure 2:**
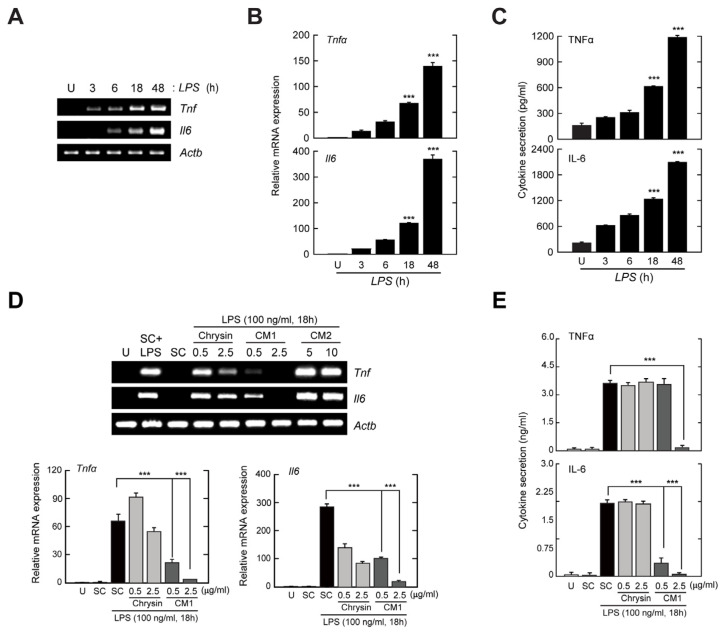
CM1 inhibits lipopolysaccharide (LPS)-induced inflammatory responses in bone marrow-derived macrophages (BMDMs). (**A**–**C**) BMDMs were incubated with LPS (100 ng/mL) for the indicated times. (**A**,**B**) mRNA levels of *Tnfα* and *Il6* analysed by reverse-transcription polymerase chain reaction (RT-PCR) and real-time PCR (qPCR). (**C**) Protein levels of TNF-α and IL-6 in culture medium measured by enzyme-linked immunosorbent assay (ELISA). (**D**,**E**) BMDMs were stimulated with LPS and co-treated with chrysin (0.5 or 2.5 µg/mL), CM1 (0.5 or 2.5 µg/mL), or CM2 (5 or 10 µg/mL) for 18 h. (**D**) mRNA levels of *Tnfα* and *Il6* evaluated by RT-PCR (top) and qPCR (bottom). (**E**) Protein levels of TNF-α and IL-6 in culture medium investigated by ELISA. Data are representative of three independent experiments and are presented as means ± SD. *** *p* < 0.001, compared with control cells (two-tailed Student’s *t*-test). U, untreated cells; SC, solvent control (0.01% DMSO).

CM1 attenuated LPS-induced inflammatory responses in primary dendritic cells [15] and RAW264.7 macrophages [16]. Therefore, we examined the anti-inflammatory effects of CM1, chrysin, and CM2 on LPS-stimulated BMDMs. We stimulated BMDMs with LPS for the indicated periods and assessed the levels of mRNA (Figure 2A,B) and protein (Figure 2C) of the pro-inflammatory cytokines TNF-α and IL-6.

To determine whether chrysin, CM1, and CM2 inhibit the TLR4-mediated production of inflammatory cytokines, BMDMs were stimulated with LPS in the presence or absence of each polyphenol flavonoid, and the expression levels of TNF-α and IL-6 were measured by reverse-transcription polymerase chain reaction (RT-PCR) (Figure 2D), quantitative PCR (qPCR) (Figure 2E), and enzyme-linked immunosorbent assay (ELISA) (Figure 2E). The LPS-induced expression levels of *Tnfα* and *Il6* were inhibited by CM1 in a concentration-dependent manner compared with chrysin (Figure 2D,E). Moreover, the levels of TNF-α and IL-6 in cell culture supernatants after LPS stimulation were reduced by CM1, but not by chrysin (Figure 2E). However, these inhibitory effects were not detected in the presence of CM2, rather, the TLR4-induced expression of *Tnfα* and *Il6* was increased by CM2 (Figure 2D). These results indicated that CM1 resulted in the greatest attenuation of TLR4-mediated production of inflammatory cytokines in primary macrophages.

### 3.3. CM1 Attenuates TLR4-Induced Activation of ERK 1/2 MAPK and the NF-κB Signalling Pathway in Primary Macrophages

**Figure 3:**
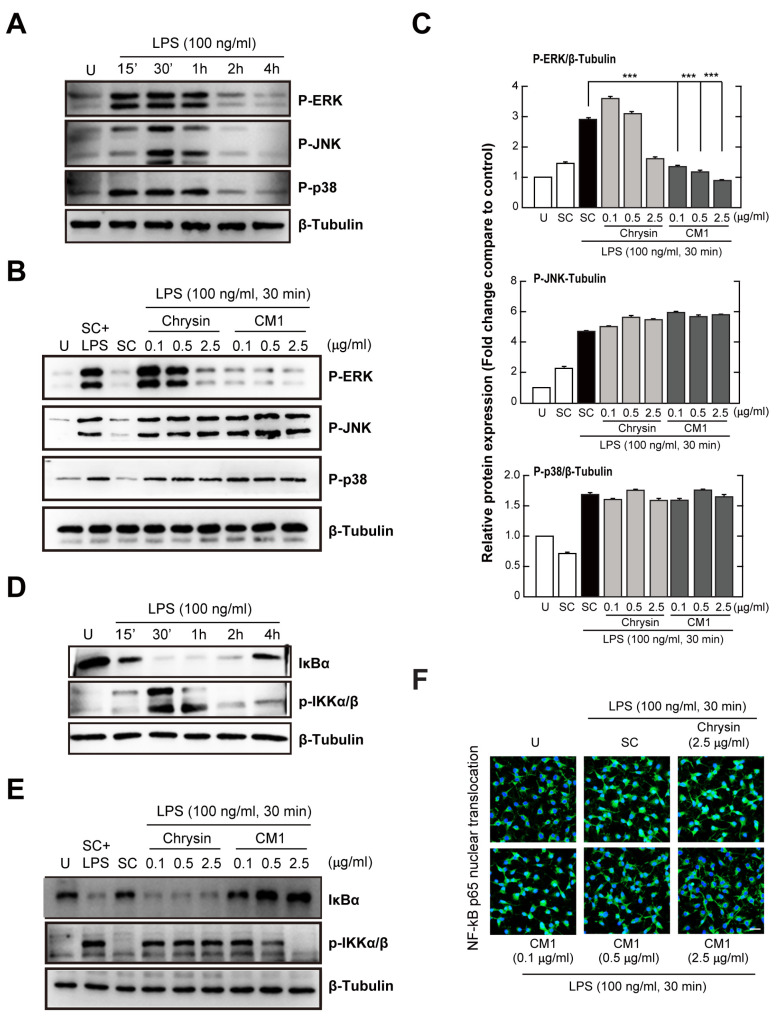
CM1 inhibits LPS-induced extracellular-signal-regulated kinase (ERK) phosphorylation and nuclear factor kappa B (NF-κB) activation. (**A**,**D**) BMDMs were incubated with LPS (100 ng/mL) for the indicated times. Mitogen-activated protein kinase (MAPK) and NF-κB activation were determined by immunoblotting. (**B**,**C**,**E**) BMDMs were stimulated with LPS only, LPS and chrysin (0.1, 0.5, or 2.5 µg/mL), or LPS and CM1 (0.1, 0.5, or 2.5 µg/mL) for 30 min. (**B**,**E**) Protein levels determined by immunoblotting. (**C**) Densitometric analysis of p-ERK, p-JNK, and p-p38 expression with normalisation to β–tubulin. (**F**) Cells were fixed and stained for NF-κB p65 (green); nuclei were stained with 4′,6-diamidino-2-phenylindol (DAPI) (blue). Nuclear translocation of NF-κB p65 analysed by confocal microscopy. Scale bar: 20 µm. Data are representative of three independent experiments and are presented as means ± SD. *** *p* < 0.001, compared with control cells (two-tailed Student’s *t*-test). U, untreated cells; SC, solvent control (0.01% DMSO).

Next, we investigated whether CM1 modulates intracellular signalling pathways related to the TLR4-mediated activation of inflammatory responses. LPS stimulation for 60 min resulted in marked activation (Figure 3A) of the three MAPK subfamilies, ERK 1/2, p38, and c-Jun N-terminal kinase (JNK), that are required for the activation of inflammatory genes upon TLR stimulation through transcription factor complex AP-1 [19]. The LPS-induced phosphorylation of ERK 1/2, but not p38 and JNK, was more attenuated by CM1 than by chrysin; in addition, the effect was concentration-dependent (Figure 3B,C).

NF-κB signalling modulates the transcription of genes that regulate TLR4-mediated inflammation [20]. Incubation with LPS (Figure 3D) led to rapid, strong activation of IKKα and IKKβ and degradation of the NF-κB inhibitor, IκB-α. To investigate the inhibitory effect of CM1 on LPS-mediated activation of NF-κB signalling, cells were incubated with LPS for 30 min in the presence of chrysin or CM1, and the activation of IKKα/β and degradation of IκB-α were evaluated by immunoblotting. CM1 attenuated the LPS-induced activation of NF-κB signalling in primary macrophages (Figure 3E). However, no such effect was noted in chrysin-treated cells. Moreover, LPS-induced nuclear translocation of NF-κB was attenuated in primary macrophages treated with CM1, but not with chrysin (Figure 3F). Together, these results showed that CM1 negatively regulated the TLR4-mediated activation of the MAPK/ERK 1/2 and NF-κB signalling pathways, which may be essential for the regulation of inflammatory responses in BMDMs.

### 3.4. TNFAIP3 Is Crucial for the CM1-Mediated Anti-Inflammatory Effect in TLR4-Stimuated Cells

**Figure 4:**
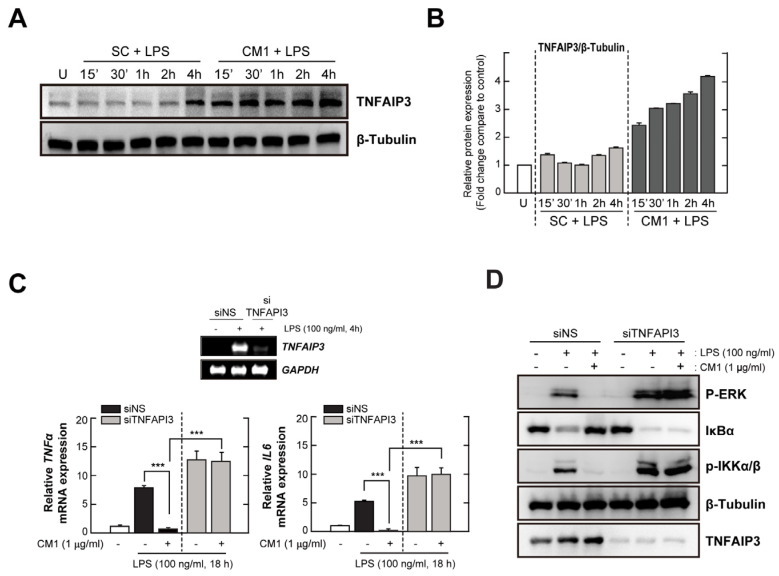
CM1 attenuates LPS-induced inflammatory responses through TNFAIP3 upregulation. (**A**,**B**) BMDMs were stimulated with LPS only (100 ng/mL) or LPS with CM1 (1 µg/mL) for the indicated times. (**A**) Protein levels of tumour necrosis factor alpha-induced protein 3 (TNFAIP3) determined by immunoblotting. (**B**) Densitometric analysis of TNFAIP3 expression with normalisation to β–tubulin. (**C**,**D**) HeLa cells were transfected with control siRNA or siTNFAIP3. (**C**) Transfected cells were stimulated with LPS only or LPS and CM1 for 18 h. mRNA levels of *Tnfα* and *IL6* were determined by qPCR. RT-PCR was performed to assess transfection efficiency (inset). (**D**) Transfected cells were co-stimulated with LPS and CM1 for 30 min. Protein levels of p-ERK, p-IKKαβ, total IκBα, and TNFAIP3 were evaluated by immunoblotting. Data are representative of three independent experiments and are presented as means ± SD. *** *p* < 0.001, compared with control cells (two-tailed Student’s *t*-test). U, untreated cells; SC, solvent control (0.01% DMSO); siNS, non-specific siRNA; siTNFAIP3, specific siRNA for TNFAIP3.

CM1 exerted an anti-inflammatory effect by upregulating Tollip in LPS-stimulated RAW264.7 macrophages [16], suggesting that it may have a potent role to activate other TLR-negative regulators. Therefore, we evaluated whether TNFAIP3 was required for the anti-inflammatory effect of CM1 in BMDMs and that of Tollip in RAW264.7 macrophages. CM1 more strongly upregulated TNFAIP3 in LPS-stimulated BMDMs compared to SC (Figure 4A,B).

To assess the role of TNFAIP3 in the anti-inflammatory effect of CM1 upon LPS stimulation, we transfected HeLa cells with an siRNA specific for TNFAIP3 (siTNFAIP3), which silenced human *TNFAIP3*, but not the housekeeping gene *GAPDH* (Figure 4C, upper). TNFAIP3 knockdown in HeLa cells abolished the CM1-mediated effect on the mRNA levels of TNF-α and IL-6, whereas CM1-treated HeLa cells transfected with a scrambled control siRNA showed reduced mRNA levels of these cytokines, as did primary macrophages (Figure 4C, bottom). Moreover, the inhibitory effect of CM1 on the LPS-induced activation of MAPK/ERK 1/2 and NF-κB signalling was abolished by TNFAIP3 knockdown in HeLa cells (Figure 4D). Collectively, these findings suggested that CM1 attenuated the LPS-mediated activation of pro-inflammatory responses by upregulating TNFAIP3.

### 3.5. CM1 Negatively Regulates TLR4-Induced Acetylation of NF-κB p65 by Activating SIRT1

**Figure 5:**
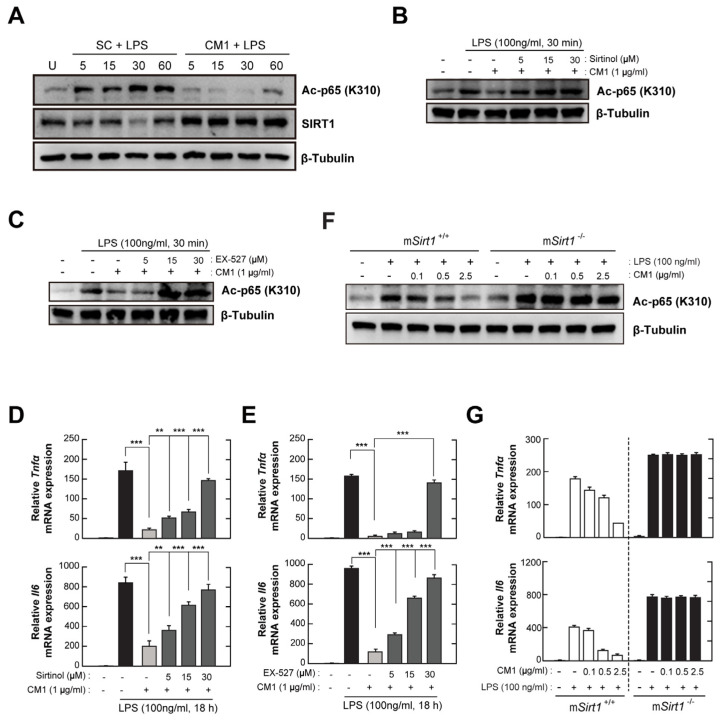
CM1 reduces LPS-induced inflammatory responses by enhancing SIRT1 activity. (**A**) BMDMs were stimulated with LPS only (100 ng/mL) or LPS and CM1 (1 µg/mL) for the indicated times. NF-κB p65 acetylation and SIRT1 expression were determined by immunoblotting. (**B**–**E**) BMDMs were pre-treated with increasing concentrations of sirtinol (5, 15, or 30 µM; B and D) or EX-527 (5, 15, or 30 µM; (**C**,**E**)) for 2 h, followed by exposure to LPS only or LPS and CM1 for 30 min (**B**,**C**) or 18 h (**D**,**E**). (**B**,**C**) Immunoblotting was performed to evaluate the acetylation of NF-κB p65. (**D**,**E**) mRNA levels of *Tnfα* and *Il6* determined by qPCR. (**F**,**G**) BMDMs from m*Sirt1^+/+^* and m*Sirt1^−/−^* mice stimulated with LPS only or LPS and CM1 (0.1, 0.5, or 2.5 µg/mL) for 30 min (for (**F**)) or 18 h (for (**G**)). (**F**) NF-κB p65 acetylation was determined by immunoblotting. (**G**) mRNA levels of *Tnfα* and *Il6* measured by qPCR. Data are representative of three independent experiments and are presented as means ± SD. ** *p* < 0.01, *** *p* < 0.001, compared with control cells (for (**D**,**E**)) or cells isolated from *Sirt1^+^*^/*+*^ mouse (two-tailed Student’s *t*-test). U, untreated cells; SC, solvent control (0.01% DMSO).

SIRT1 is required for the activation of protective immune responses, and impairment of SIRT1 results in the progression of autoimmune and inflammatory diseases [8]. Moreover, the activation of SIRT1 by naturally occurring dietary polyphenols is implicated in the regulation of oxidative stress, inflammation, and autoimmunity in response to endogenous or exogenous stimuli [21]. CM1 increased SIRT1 expression and decreased NF-κB p65 acetylation in LPS-stimulated BMDMs (Figure 5A). However, the effect of CM1 on LPS-mediated NF-κB p65 acetylation was abolished by pre-treatment with sirtinol (Figure 5B) or EX-527 (Figure 5C), which are selective inhibitors of SIRT1. Additionally, the effect of CM1 on the mRNA levels of TNF-α and IL-6 was impaired by sirtinol (Figure 5D) and EX-527 (Figure 5E).

To assess the role of myeloid-specific SIRT1 in the anti-inflammatory effect of CM1, BMDMs from m*Sirt1*^+/+^ or m*Sirt1*^−/−^ mice were stimulated with LPS in the presence of the indicated concentrations of CM1 (Figure 5F,G). LPS-mediated NF-κB p65 acetylation in m*Sirt1*^+/+^ BMDMs was attenuated by CM1 in a concentration-dependent manner; however, the deficiency of myeloid-specific SIRT1 impaired the CM1-mediated de-acetylation of NF-κB p65 (Figure 5F), similar to selective SIRT1 inhibitors (Figure 5B,C). Consistently, the CM1-mediated anti-inflammatory effect was abolished in *Sirt1*-deficient BMDMs, compared to m*Sirt1*^+/+^ BMDMs. These results indicate that myeloid-specific SIRT1 is crucial for the inhibitory effect of CM1 on TLR4-induced inflammation in primary murine macrophages.

### 3.6. CM1 Prevents Systemic Inflammation In Vivo, Leading to Decreased Mortality during Endotoxin-Induced Lethal Shock

**Figure 6:**
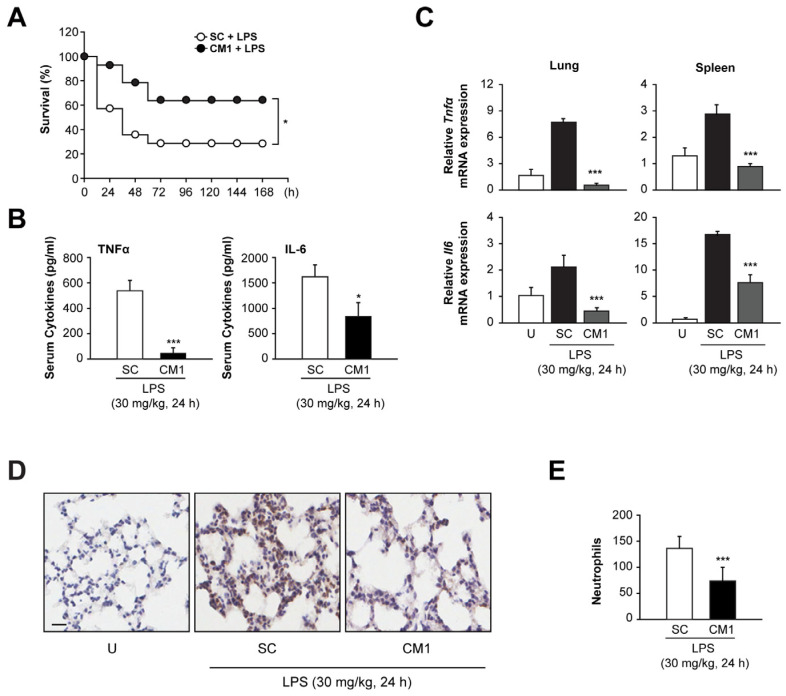
CM1 contributes to the protection of mice against lethal shock. (**A**–**E**) Mice (*n* = 10 per group) were intravenously injected with either vehicle control or CM1 (10 mg/kg) once daily for 3 days before endotoxin stimulation (intraperitoneal injection, 30 mg/kg). (**A**) Survival rates of each group were monitored for 168 h. (**B**–**E**) Mice were sacrificed at 24 h post-LPS injection (*n* = 5 per group). (**B**) Serum samples were collected from vehicle control-treated or CM1-treated mice. Levels of TNFα and IL-6 were determined using ELISA. (**C**) The expression of *Tnfα* and *Il6* in lung (left) and spleen (right) was analysed using real-time qPCR. (**D**) Immunohistochemical analysis of the lung tissue was performed to determine neutrophil infiltration. Scale bar: 50 µm. (**E**) The number of infiltrating neutrophils was counted from 8 random fields. The experiments were conducted in triplicate to ensure reproducibility, with the results expressed as the means ± SD. Statistical significance of mean differences was determined using either a log-rank test (**A**) or a two-tailed Student’s *t*-test (**B**,**C**,**E**). * *p* < 0.05, *** *p* < 0.001, compared with control mice stimulated with LPS. U, untreated; SC, solvent control.

We evaluated the cytotoxicity of CM1. CM did not affect the feeding, drinking, or hair colour of mice. Moreover, body weight (Figure S1) and haematological and serum biochemical markers (Table S1) were not altered by CM1. To examine the in vivo effect of CM1, we used mice with endotoxin-induced septic shock. Endotoxin-induced lethality was significantly decreased in CM1-treated mice compared with SC-treated mice (Figure 6A). Consistently, the serum levels of TNF-α and IL-6 were significantly decreased in CM1-treated mice after LPS challenge (Figure 6B). Next, we evaluated the mRNA levels of pro-inflammatory cytokines in lung and spleen tissues after LPS challenge. The endotoxin-induced expression of TNF-α and IL-6 was significantly attenuated in the lung and spleen tissues of CM1-treated mice compared with SC-treated mice (Figure 6C). In addition, neutrophil infiltration into lung tissues was reduced in CM1-treated mice compared with SC-treated mice after LPS challenge (Figure 6D,E). These results suggest that CM1 prevents lethality by regulating systemic inflammation in mice with septic shock.

## 4. Discussion

**Figure 7:**
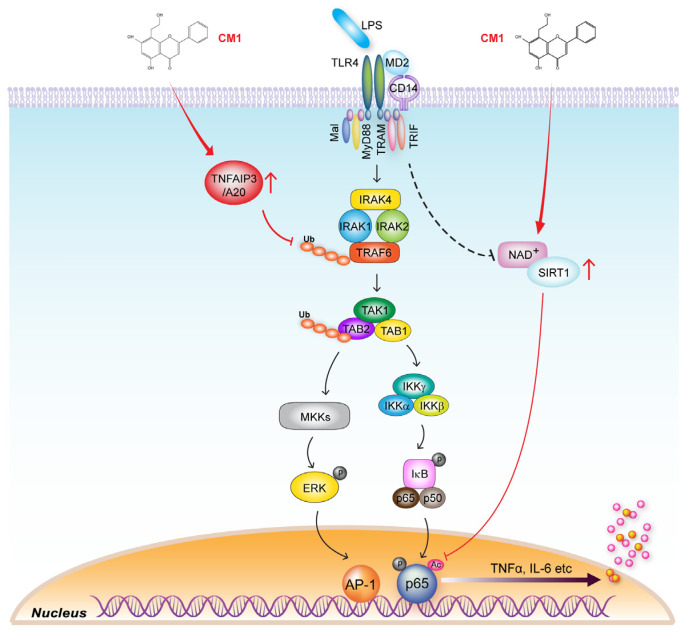
Inhibitory effect of CM1 on TLR4-induced inflammation and its molecular mechanisms. Upon the binding of LPS to the TLR4/MD2/CD14 complex, the downstream signalling cascade is activated, leading to the ubiquitination of TRAF6 and subsequently activation of TAK1 and the IKK complex, which phosphorylates IκB, leading to the release of NF-κB p65 into the nucleus. This process results in the transcription of pro-inflammatory cytokines such as TNF-α and IL-6. CM1 directly upregulates the expression of TNFAIP3/A20, which inhibits the NF-κB p65 pathway and enhances the activity of SIRT1. Increased SIRT1 activity, in turn, deacetylates p65, suppressing its ability to transcribe pro-inflammatory genes. The dual action of CM1 suggests its therapeutic potential for LPS-induced inflammation.

We have reported that CM1 exerts an anti-inflammatory effect on TLR4-stimulated primary dendritic cells and mice with DSS-induced colitis and has therapeutic potential for inflammatory bowel disease [15]. Moreover, CM1 strongly induced the expression of Tollip in macrophages, thereby strongly suppressing TLR4-mediated inflammation [16]. These findings suggested that CM1 modulates the expression of other negative regulators during TLR4 stimulation. In the present study, we demonstrated that CM1 negatively regulates TLR4-induced hyper-activation of inflammatory responses in primary macrophages in vitro via TNFAIP3-mediated regulation of the ERK/MAPK NF-κB signalling pathway by inhibiting TRAF6 ubiquitination and post-translational modification by means of SIRT1-mediated de-acetylation of NF-κB p65 (Figure 7). In addition, CM1 increased the survival rate and decreased inflammatory responses in vivo in a mouse model of sepsis.

TLRs are innate immune receptors that recognise infectious agents and their ligands, resulting in the activation of intracellular signalling cascades that initiate inflammatory responses [20]. Inflammation is crucial for host protection against microbial infections; however, excessive and uncontrolled inflammatory responses are harmful and can lead to multiple organ dysfunction and failure [22]. In this study, CM1 suppressed the LPS-mediated generation of TNF-α and IL-6 (Figure 2). In vivo, CM1 reduced systemic inflammation and lethality in mice with endotoxin-induced septic shock (Figure 6). In addition, LPS stimulation resulted in the activation of NF-κB and ERK/MAPK signalling, which was abolished by CM1. However, the LPS-induced activation of p38 and JNK was not affected by CM1 (Figure 3). These results partly correlate with our prior findings and show that LPS-induced MAPK activation was attenuated by CM1 in RAW 264.7 macrophages [16] and primary dendritic cells [15]. Therefore, the mechanism of action of CM1 may differ depending on the cell type.

TLR signalling pathways are controlled by multiple steps through the transcriptional, epigenetic and post-translational regulation of diverse target genes [23]. Indeed, multiple negative regulators of host inflammatory and immune responses have been reported [24]. TRAF6, an essential adaptor molecule in TLR signalling, is targeted by several negative regulators, including TNFAPI3, oestrogen-related receptor α, cylindromatosis, small heterodimer partner, and TANK [7,10,25,26,27,28,29]. Among these, TNFAIP3, which is also known as A20, is a multifunctional protein with E3 ubiquitin ligase and deubiquitinase activities and terminates TLR-induced NF-κB and MAPK signalling by regulating the K63-linked polyubiquitination of TRAF6 [7,30]. Chrysin exerts an anti-neuroinflammatory effect in response to LPS in vitro in BV2 and primary microglial cells and in vivo, an effect mediated by the inhibition of TRAF6 polyubiquitination and the NF-κB pathway via TNFAIP3 upregulation [31]. However, the involvement of TNFAIP3 in the anti-inflammatory effect of CM1 has remained unclear. In this study, we found that TNFAIP3 production was induced within 15 min after co-treatment with CM1 and LPS and was maintained for up to 4 h in primary macrophages. Its production began earlier, reached a higher level, and was maintained for longer than in cells stimulated with LPS alone (Figure 4A,B). In addition, siRNA-mediated knockdown of TNFAIP3 in HeLa cells abolished the inhibitory effect of CM1 on the LPS-mediated production of inflammatory cytokines and the activation of NF-κB and ERK/MAPK signalling (Figure 4C,D). These results indicated that TNFAIP3 is essential for the anti-inflammatory effect of CM1 in response to TLR4. Future studies should identify the molecular mechanisms by which CM1 increases TNFAIP3 expression and examine its effect on enzymatic activity.

SIRT proteins act as NAD^+^-dependent deacetylases and link infection and inflammation in human health and disease [9,32,33,34,35]. During inflammation, SIRT1 deacetylates NF-κB p65 at lysine310 and promotes its translocation to the cytoplasm from the nucleus, leading to the inactivation of NF-κB signalling and termination of inflammatory responses [36]. SIRT1-deficient mice show increased NF-κB acetylation and activation, exacerbating pulmonary vascular leakage and lung inflammation after exposure to particulate matter [37]. Myeloid-specific SIRT1 activates the protective autophagic response against *Toxoplasma gondii* infection by controlling the deacetylation of FoxO1 and FoxO3a [34]. Chrysin was found to protect against carrageenan-induced pulmonary and pleural injury in rats and attenuated the activation of neutrophils and oxidative stress by regulating the activity of NF-κB via the SIRT1/NRF2 signalling pathway [38], suggesting that CM1 regulates excessive inflammation by modulating SIRT1 signalling. In this study, the pharmacological inhibition of SIRT1 resulted in, and myeloid-specific SIRT1-deficient macrophages demonstrated, the abrogation of the anti-inflammatory effect of CM1. Our findings indicate that CM1 regulates the SIRT1-mediated deacetylation of NF-κB p65, thereby affecting inflammatory responses in primary macrophages (Figure 5).

## 5. Conclusions

CM1 exerted an anti-inflammatory effect during endotoxin stimulation by regulating the NF-κB and ERK/MAPK signalling pathways via the upregulation of TNFAPI3 and SIRT1. Although challenges remain, including the mass synthesis of CM1, in vivo toxicity testing, and human studies, we believe that our findings will facilitate the development of novel therapeutics to control inflammation-associated diseases.

## Data Availability

Data are contained within the article and Supplementary Materials.

## Supplementary Materials

The following supporting information can be downloaded at: https://www.mdpi.com/article/10.3390/nu16050641/s1, Figure S1. Monitoring of body weight in mice received with solvent control or CM1. Table S1. Monitoring of hematology and serum biochemical markers in mice received with solvent control or CM1.
