# Supplementary material for: CM1, a Chrysin Derivative, Protects from Endotoxin-Induced Lethal Shock by Regulating the Excessive Activation of Inflammatory Responses
Source: Nutrients. 2024 Feb 25;16(5):641. doi: 10.3390/nu16050641 (PMC10934210; doi:10.3390/nu16050641)
Supplement: Supplementary file 1 [file nutrients-16-00641-s001.zip › nutrients-2824476-supplementary.pdf]

nutrients

Supplementary Materials

CM1, a Chrysin Derivative, Protects from Endotoxin-Induced Lethal Shock by Regulating the Excessive Activation of Inflammatory Responses

Jae-Hyung Lee [1,2,3,†], Young-Bok Ko [3,4,†], Yong-Min Choi [1,2,3], Jinju Kim [1,2,3], Hwan-Doo Cho [1,2], Hyeonil Choi [1,2], Ha-Yeon Song [5], Jeong-Moo Han [5], Guang-Ho Cha [1,2,3], Young-Ha Lee [1,2,3], Jin-Man Kim [2,3,6], Woo-Sik Kim [7,*], Eui-Baek Byun [5,*] and Jae-Min Yuk [1,2,3,*]

[1] Department of Infection Biology, College of Medicine, Chungnam National University, Daejeon 35015, Republic of Korea; dlwogud1003@naver.com (J.-H.L.); ymcdragon@naver.com (Y.-M.C.); w1l2s3@naver.com (J.K.); johwandoo@naver.com (H.-D.C.); perfectviewman@naver.com (H.C.); gcha@cnu.ac.kr (G.-H.C.); yhalee@cnu.ac.kr (Y.-H.L.)
[2] Infection Control Convergence Research Center, College of Medicine, Chungnam National University, Daejeon 35015, Republic of Korea; jinmank@cnu.ac.kr
[3] Department of Medical Science, College of Medicine, Chungnam National University, Daejeon 35015, Republic of Korea; koyoung27@gmail.com
[4] Department of Obstetrics & Gynecology, Chungnam National University Hospital, Daejeon 35015, Republic of Korea
[5] Korea Advanced Radiation Technology Institute, Korea Atomic Energy Research Institute, Jeongeup 56212, Jeonbuk, Republic of Korea; songhy@kaeri.re.kr (H.-Y.S.); jmhahn@kaeri.re.kr (J.-M.H.)
[6] Department of Pathology, College of Medicine, Chungnam National University, Daejeon 35015, Republic of Korea
[7] Functional Biomaterial Research Center, Korea Research Institute of Bioscience and Biotechnology, Jeongeup 56212, Jeonbuk, Republic of Korea
* Correspondence: kws6144@kribb.re.kr (W.-S.K.); ebbyun80@kaeri.re.kr (E.-B.B.); yjaemin0@cnu.ac.kr (J.-M.Y.)
† These authors contributed equally to this work.

Supplemental Materials and methods

Routine Blood Examination and Serum Biochemistry

After administration of solvent control or CM1, blood samples from each group were obtained on day 3. Routine blood examination was conducted using a blood analyzer (IDEXX, Procyte Dx, USA). Serum biochemistry were measured by a blood biochemistry analyzer (IDEXX, CatalystOne, USA).

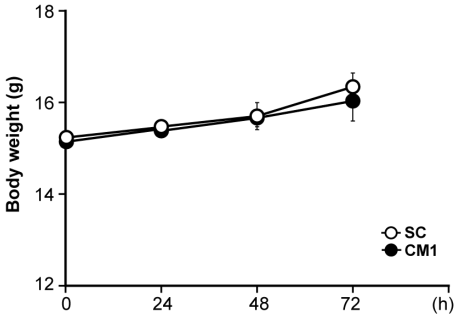

Figure S1. Monitoring of body weight in mice received with solvent control or CM1. Mice (n = 5 per group) were intravenously injected with either vehicle control or CM1 (10 mg/kg) once daily for 3 days and the body weight then was monitored for 3 days. SC, solvent control.

Table S1. Monitoring of hematology and serum biochemical markers in mice received with solvent control or CM1. Mice (n = 5 per group) were intravenously injected with either vehicle con-

trol or CM1 (10 mg/kg) once daily for 3 days and the blood of mice then was collected. Hematology markers and serum biochemical markers were analyzed using a routine blood examination and clinical biochemistry examination, respectively. SC, solvent control; RBC, red blood cell; HCT, hematocrit value; HB, hemoglobin level; MCV, mean corpuscular volume; MCH, mean corpuscular hemoglobin; MCHC, mean corpuscular hemoglobin concentration; WBC, white blood cell.

**Marker**	**SC**	**CM1**	***p*-Value**
**Hematology marker**			
RBC ($\times 10^{12}$/L)	8.24 ± 0.54	8.13 ± 0.53	0.78608
HCT (%)	40 ± 2.62	39.6 ± 2.24	0.848472
HB (g/dL)	12 ± 0.79	11.9 ± 0.72	0.856374
MCV (fL)	48.5 ± 0.57	48.7 ± 1.28	0.740693
MCH (pg)	14.5 ± 0.14	14.6 ± 0.19	0.523742
MCHC (g/dL)	29.9 ± 0.15	29.9 ± 0.44	0.933529
WBC ($\times 10^{9}$/L)	0.76 ± 0.12	0.61 ± 0.13	0.31308
Neutrophil (%)	3.13 ± 0.97	7 ± 2.32	0.097733
Lymphocyte (%)	71.4 ± 7.28	78.5 ± 5.12	0.326314
Monocyte (%)	16 ± 3.36	18.9 ± 6.4	0.677637
Basophil (%)	3.77 ± 0.66	4.8 ± 0.6	0.262215
**Serum biochemical marker**			
ALT (U/L)	65.3 ± 4.5	64.7 ± 0.94	0.847438
AST (U/L)	249.3 ± 39.6	229 ± 35.2	0.616322
